# The learning curve for intraoperative neuromonitoring of the recurrent laryngeal nerve in thyroid surgery

**DOI:** 10.1007/s00423-016-1438-8

**Published:** 2016-05-13

**Authors:** Beata Wojtczak, Krzysztof Kaliszewski, Krzysztof Sutkowski, Mateusz Głód, Marcin Barczyński

**Affiliations:** 1 Department and Clinic of General, Gastroenterological and Endocrine Surgery, Wroclaw Medical University, Wroclaw, Poland; 2 Department of Endocrine Surgery, Third Chair of General Surgery, Jagiellonian University Medical College, Kraków, Poland

**Keywords:** Learning curve, Thyroid surgery, IONM, Recurrent laryngeal nerve

## Abstract

**Purpose:**

Intraoperative neuromonitoring (IONM) of the recurrent laryngeal nerve (RLN) is often used in thyroid surgery. However, this procedure is complex and requires a learning period to master the technique. The aim of the study was to evaluate the learning curve for IONM.

**Methods:**

A 3-year period (2012–2014) of working with IONM (NIM3.0, Medtronic) was prospectively analyzed with a special emphasis on comparing the initial implementation phase in 2012 (101 patients, 190 RLNs at risk) with subsequent years of IONM use in 2013 (70 patients, 124 RLNs at risk) and 2014 (65 patients, 120 RLNs at risk).

**Results:**

The rate of successful IONM-assisted RLN identification increased gradually over the 3-year study period (92.11 % in 2012 vs. 95.16 % in 2013 vs. 99.16 % in 2014; *p* = 0.022), with a corresponding decrease in the rate of technical problems (12.87, 4.3, and 4.6 %, respectively; *p* = 0.039). The rate of RLN injuries tended to decrease over time: 3.68, 1.55, and 0.83 %, respectively (*p* = 0.220). Between 2012 and 2014, increases in the sensitivity (71.4 vs. 100 %), specificity (98 vs. 99 %), positive predictive value (62.5 vs. 75 %), negative predictive value (98 vs. 100 %), and overall accuracy of IONM (97.4 vs. 99.6 %) were observed (*p* = 0.049). Increasing experience with IONM resulted in more frequent utilization of total thyroidectomy (92 % in 2012 vs. 100 % in 2013–2014; *p* = 0.004).

**Conclusions:**

There was a sharp decrease in the number of technical problems involving equipment setup from 2012 to 2014.

## Introduction

Identification of the recurrent laryngeal nerve (RLN) during thyroid surgery minimizes the risk of nerve injury and is considered the gold standard [1–6]. Intraoperative neuromonitoring (IONM), a complement to visualization of the RLN, was introduced in 1966 by Shedd [1, 7]. It not only facilitates nerve identification but also permits intraoperative evaluation and prediction of RLN function [1, 7]. IONM is currently a standardized technology, and its everyday use is increasingly widespread [1]. Introducing this technique requires previous experience in thyroid surgery, as well as theoretical preparation, preferably at a center with extensive experience working with IONM technology. It has been found that 95 % of surgeons who have had training in IONM admitted that before undergoing an introductory course in this technique, their understanding of and work with IONM was not adequate [8]. While a course in neuromonitoring alone provides a good basis for introducing the technique for thyroid surgery, only independent experience of a number of thyroid procedures using IONM can enable surgeons to use it to its full advantage in order to minimize the RLN injury rate [8–11]. The aim of this study was to evaluate the learning curve for IONM of the RLN at an academic center.

## Material and methods

A total of 236 patients who underwent thyroid surgery with IONM at the Department of General, Gastroenterological, and Endocrine Surgery at Wroclaw Medical University in Wroclaw, Poland, between January 2012 and December 2014, were enrolled in the study. The aim of the study was to evaluate the learning curve for IONM, so a 3-year period working with IONM was prospectively analyzed with a particular emphasis on comparing the initial implementation phase in 2012 (101 patients, 190 RLNs at risk) with subsequent years of IONM use: 2013 (70 patients, 124 RLNs at risk) and 2014 (65 patients, 120 RLNs at risk). The outcomes of this study were the rate of successful RLN identification, the rate of equipment setup problems, the rate of RLN injury, and the duration of surgery with IONM. In addition, intraoperative loss of signal; the positive and negative predictive value of IONM; and its sensitivity, specificity, and accuracy were also assessed. The study was approved by the Bioethics Committee of Wroclaw Medical University.

The patients’ demographic and intraoperative data is presented in Table 1. All the patients included in the study were screened preoperatively by an endocrinologist; the only criteria for exemption from the study were abnormal thyroid hormone levels before surgery and vocal cord paralysis found in the preoperative laryngological examination. In order to confirm the participants’ euthyroid status, in addition to the standard preoperative tests, each patient’s TSH and FT4 were determined immediately before surgery. The study participants included patients with benign goiter as well as those with thyroid cancer, and the surgery performed included both primary thyroid procedures and reoperations.

**Table 1:** Demographic and intraoperative characteristics of the 236 patients included in the study

	IONM implementation phase (2012)	IONM—the subsequent phase (2013–2014)	*p* value
Patients, no.	101	135	–
RLNs at risk, no.	190	244	–
Sex ratio (F/M)	4.9	4.4	0.864^a^
Mean age ± SD, years	54.99 ± 13.08	53.24 ± 13.55	0.319^b^
Primary procedures, no. (%)	92 (91.09)	107 (79.26)	0.018^a^
Secondary procedures, no. (%)	9 (8.91)	28 (20.74)	
Nodular goiter, no. (%)	74 (73.27)	92 (68.15)	0.472^a^
Toxic nodular goiter, no. (%)	10 (9.9)	16 (11.85)	0.680^a^
Grave’s disease, no. (%)	4 (3.96)	5 (3.7)	0.986^a^
Thyroid carcinoma, no. (%)	13 (12.87 %)	22 (16.3 %)	0.579^a^
Volume of the goiter ± SD, ml	45.56 ± 39.87	37.94 ± 30.04	0.177^c^
Retrosternal goiter, no. (%)	25 (24.75)	27 (20.00)	0.526^a^
Compression or narrowing of tracheae, no. (%)	59 (58.42)	70 (51.85)	0.356^a^

*p* Value <0.05 was considered statistically significant

*RLN* recurrent laryngeal nerve, *ns* non significant

^a^Fisher’s test

^b^ *t* test

^c^Mann-Whitney test

All the thyroid procedures were performed by a team of three surgeons (average age 41), who annually perform 50–100 thyroid operations. Before the initial implementation of IONM in thyroid surgery, both the surgical team and the anesthesiology team underwent introductory training in the technique at the Department of Endocrine Surgery at Jagiellonian University Medical College in Krakow, Poland. For the team of surgeons, it was the first experience working with IONM. RLN monitoring was carried out in accordance with the recommendations of the International Nerve Monitoring Study Group [1], using NIM-3.0 equipment (Medtronic, Jacksonville, USA). A handheld monopolar stimulating probe was used for nerve stimulation with a current amplitude of 1 mA (range 0.5–1.5 mA) and 3-Hz impulses of 200 ms each for 1–2 s. The electromyographic (EMG) signal was obtained using surface electrodes integrated in the endotracheal tube (NIM Flex EMG tube, Medtronic). In female patients, 7-mm endotracheal tubes were used; in males, 7.5- or 8-mm tubes were used. The endotracheal tube was placed about 20–22 cm from the incisors, with particular emphasis on the precise positioning of the electrodes between the vocal folds.

Every patient underwent a laryngological examination to assess vocal cord mobility (L1). Before dissection, neuromonitoring was initiated by vagal stimulation (V1) on the operated side of the thyroid. Before removing the thyroid lobe, the RLN was identified or mapping techniques were used to find it (R1). Then, after the removal of the thyroid lobe, RLN conduction (R2) and the whole path of reflex, including vagal nerve conduction, was confirmed (V2).

Postoperative laryngoscopy was routinely used to diagnose and follow up RLN injury. On the first postoperative day, an ENT specialist performed a functional assessment of the larynx by indirect laryngoscopy. Patients with postoperative dysfunction of the vocal cords were evaluated by videolaryngoscopy up to 6 months postoperatively to assess the mobility of their vocal cords. Cases in which the function of the vocal cords was recovered within 6 months were considered transient paresis, while a lack of vocal cord mobility 6 months after the operation was considered permanent palsy.

Both the number of RLNs identified and the number of nerve injuries were assessed in relation to the number of RLNs at risk of injury. An evaluation of IONM was carried out using the definitions presented by Chang and Lo [12]. Technical problems were assessed in relation to the number of thyroid procedures performed with the use of neuromonitoring.

A statistical analysis was carried out to evaluate the learning curve for IONM, using Prism 5.0 statistical software (GraphPad, La Jolla, USA) to analyze the data. Relationships among the clinicopathological parameters were analyzed by Fisher’s exact test and the *χ* ^2^ test. To compare parametric data, the unpaired Student’s *t* test was used, while the Mann-Whitney *U* test was used to compare groups of data which did not meet the assumptions of the parametric test. To compare more than two groups, the Kruskal-Wallis test was applied, with a post hoc analysis using Dunn’s multiple comparison test. To assess the diagnostic accuracy of IONM, received operating characteristic (ROC) curves were analyzed. In all the analyses, results were considered statistically significant when *p* < 0.05.

## Results

In 2012, out of 190 RLNs at risk, 175 (92.11 %) were identified with IONM. In subsequent years, an increase in RLN identification with IONM was observed: In 2013, out of 124 RLNs at risk, 118 (95.16 %) were identified, and in 2014, out of 120 RLNs at risk, 119 (99.16 %) were identified. The increases in RLN identification between successive years—2012–2013 and 2013–2014—were not at the level of statistical significance (*p* > 0.05) according to Fisher’s exact test, but considering the entire 3-year period working with IONM, the increase was at the level of statistical significance (*p* = 0.022) according to the chi-square test.

There was a sharp decrease in the number of technical problems involving equipment setup from 2012 to 2014 (*p* = 0.039) and between the initial implementation phase (2012) and the subsequent phase (2013–2014) (*p* = 0.019). The biggest decline was observed after the first 100 thyroid operations in 2012 (12.87 %); subsequently, the frequency of technical problems remained at more or less the same level: 4.3 % in 2013 and 4.6 % in 2014. During the 3-year period working with IONM (236 operations), problems related to the surface electrodes on the intubation tube (7.2 %) were more frequent than problems involving the grounding electrodes (0.85 %). The most common recording-side problems were malpositioning of the endotracheal tube (5.08 %), the tube being inserted too deep (1.27 %), and the tube size not fitting the patient adequately (0.84 %.). Slippage of the grounding electrode (0.85 %) was observed only during the first year of IONM use. Details of the technical problems with IONM in the initial implementation phase (2012) vs. the subsequent phase (2013–2014) are shown in Table 2. The largest decrease in technical problems between 2012 and 2013–2014 was related to endotracheal tube rotation: 7.92 vs. 2.96 %.

**Table 2:** Thyroid operations with IONM—equipment setup problems

Technical problems	Implementation phase: 2012	Subsequent phase: 2013–2014
	*n* = 101 operations	*n* = 135 operations
Problems involving surface electrodes on the intubation tube	11 (10.89 %)	6 (4.44 %)
Endotracheal tube rotation	8 (7.92 %)	4 (2.96 %)
Inadequate endotracheal tube	1 (0.99 %)	1 (0.74 %)
Endotracheal tube inserted too deep	2 (1.98 %)	1 (0.74 %)
Problems involving grounding electrodes	2 (1.98 %)	0 (0)
Slippage of the ground electrode	2 (1.98 %)	0 (0)
Total	13 (12.87 %)	6 (4.44 %)
Chi-square test	*p* = 0.019	

*p* Value <0.05 was considered statistically significant

*ns* non significant

Interpretations of intraoperative loss of signal are detailed in Table 3, along with the sensitivity, specificity, and accuracy of the method and its positive and negative predictive values. In the initial phase of IONM use, we observed signal loss in eight patients. In three cases, there was a false positive signal—a negative signal—but after the operation, there was no vocal cord palsy (L2). Among five patients with true positive signals, segmental loss of signal (LOS type 1) occurred in two procedures and global loss of signal (LOS type 2) occurred in three operations. In the cases of LOS type 1, the nerve was inadvertently cut in one case and crushed in the other case. Excessive traction was the main cause of LOS type 2. In the later phase of IONM use (2013–2014), there were four instances of LOS; three of these were true loss of signal, caused by traction. There was a decrease in the total rate of RLN paralysis in the early postoperative period; a descending trend was also observed in both transient and permanent nerve injury, although the decrease was not at the level of statistical significance (*p* > 0.05). Detailed data are shown in Table 4.

**Table 3:** Intraoperative loss of signal and its predictive value

IONM prognostic value	V2 signal/L2 mobility of ipsilateral vocal fold	IONM implementation phase: 2012	Subsequent IONM phase: 2013–2014	*p* value (chi-square test)
		RLNs at risk *n* = 190	RLNs at risk *n* = 244	
TN	V2(+) L2(+)	180	240	–
FN	V2(+) L2(−)	2	0	–
TP	V2(−) L2(−)	5	3	–
FP	V2(−) L2(+)	3	1	–
Sensitivity		71.4 %	100 %	*p* = 0.301
Specificity		98.3 %	99.6 %	*p* = 0.196
Positive predictive value		62.5 %	75 %	*p* = 0.665
Negative predictive value		98.9 %	100 %	*p* = 0.103
Accuracy		97.4 %	99.6 %	*p* = 0.049

*p* Value <0.05 was considered statistically significant

*IONM* intraoperative neuromonitoring, *RLN* recurrent laryngeal nerve, *TN* true negative results, *FN* false negative results, *FP* false positive results, *TP* true positive results, *V2(+)* vagal nerve signal preserved, *V2(−)* vagal nerve loss of signal, *L2(+)* normal mobility of ipsilateral vocal fold, *L2(−)* lack of mobility of ipsilateral fold

**Table 4:** Prevalence of postoperative RLN injury

	IONM 2012 (*n* = 101 operations)	IONM 2013–2014 (*n* = 135 operations)	*p* value (Fisher’s exact test)
RLN at risk (%)	190 (100)	244 (100)	–
Overall RLN paresis, no. (%)	7 (3.68)	3 (1.22 %)	*p* = 0.1124 (ns)
Transient RLN paresis, no. (%)	5 (2.63)	3 (1.22 %)	*p* = 0.3059 (ns)
Permanent RLN paresis, no. (%)	2 (1.05)	0 (0.00)	*p* = 0.1911 (ns)

*p* Value <0.05 was considered statistically significant

*IONM* intraoperative neuromonitoring, *RLN* recurrent laryngeal nerve, *ns* non significant

Increasing experience with IONM resulted in more frequent utilization of total thyroidectomy. Radical procedures (lobectomy, thyroidectomy, and near-total resection) comprised 92 % of thyroid operations in 2012; in 2013 and 2014, radical procedures constituted 100 % of all thyroid operations employing IONM. The increase in radical procedures and drop in subtotal surgeries from 2012 to 2014 was statistically significant according to the chi-square test (*p* = 0.004).

The average duration of thyroid operations with IONM in 2012 was 105 min (±31.44); in 2013, it was 118 min (±26.28); and in 2014, it was 115 min (±34.49). The Kruskal-Wallis test showed a significant difference in the distribution of the operation duration for the entire 2012–2014 period (*p* = 0.005), for 2012–2013 (*p* < 0.05), and when comparing 2012 with 2014 (*p* < 0.05). There was no difference in distribution between 2013 and 2014 (*p* > 0.05). To verify the differences between pairs of years, a test of multiple comparisons was used. Spearman’s correlation test found a statistically significant correlation (*p* = 0.004) between the patients’ weight and the duration of the operation: Higher BMIs increased the operating time, resulting in an increase of about 90–120 min for the whole range of variation.

## Discussion

The introduction of neuromonitoring to thyroid surgery made it possible not only to easily identify the RLN but also to understand the physiology and mechanisms of RLN injury [1, 6, 13–16]. Since neuromonitoring of the RLN and the external branch of the superior laryngeal nerve was standardized, the use of this technique has become increasingly widespread [1, 17]. In 2011, the first conference of the Polish Research Group for Neuromonitoring of the Polish Club of Endocrine Surgeons was held and the need to use IONM in at least some selected thyroid operations was agreed upon [18]. Since then, there has been a slow but steady growth of interest in this technique in Poland, and the number of centers performing operations with IONM continues to grow [11].

However, the introduction of IONM to thyroid surgery requires previous experience in thyroid surgery, as well as training, both theoretical and practical, in neuromonitoring [8, 9, 11, 19]. It also requires independent experience of a number of thyroid operations with IONM to improve one’s surgical technique and to reduce the number of postoperative complications [20, 21]. Pragacz et al. also reported how important this kind of training was before deploying IONM in everyday clinical practice [11, 19].

Despite solid preparation for the introduction of neuromonitoring in thyroid surgery, the authors did not entirely avoid problems during the initial stage of IONM use. These were primarily technical problems, which arose not from anything difficult about the use of the equipment but from a lack of experience with it. In the first year working with IONM (2012), problems occurred in 12.87 % of the thyroid operations, but in the next year (2013) there was already a sharp drop to 4.3 %, which was maintained at nearly the same level (4.6 %) in 2014 (*p* = 0.0219). The most common technical problem throughout the study period was rotation of the electrodes on the endotracheal tube in relation to the vocal cords. Especially during the initial phase of IONM use, many of the technical problems require knowledge of the algorithms for monitoring tube placement during intubation and problem solving in instances of signal loss. The International Nerve Monitoring Study Group recommendations for tube placement and for troubleshooting were adhered to [1]. To avoid inadequate endotracheal positioning, patients were intubated with the largest endotracheal tube possible to optimize electrode contact with the vocal cords—in women this was usually size 7.0, and in men size 7.5. After intubation and patient positioning, but before the start of surgery, preoperative tube position verification testing was carried out. We usually used the “tap test,” and in most patients this test was accurate. Whenever there were any problems or doubts, a repeat laryngoscopy was done. At the beginning of each operation, predissection vagal stimulation was the most important test to confirm overall system function. Moreover, knowledge of the standards for interpreting intraoperative loss of signal is essential when using IONM. In cases where EMG activity was not present or the amplitude was below 100 uV, the laryngeal twitch was assessed. If the laryngeal twitch was present, we suspected a malfunction on the recording side; usually, it was due to malpositioning of the endotracheal tube electrodes. Malpositioning usually involves tube rotation but may also entail inadequate tube depth. In all cases of malpositioning, a maneuver correcting the endotracheal tube placement was done by the surgeon or anesthesiologist. In two operations, we observed that the grounding electrode had slipped out of place due to perspiration. To prevent this problem, we always affixed this electrode very carefully at the beginning of surgery. We used contralateral vagal assessment in instances of signal loss, especially in bilateral surgery. If the contralateral EMG signal from the vagus nerve was absent, we checked for rotation of the tube. If the laryngeal twitch was absent or if we had the contralateral EMG signal, we suspected true loss of signal. We did not observe any intraoperative RLN stimulation errors.

A similar proportion of technical problems (10 %) was described by Dionigi et al. in those authors’ first 152 operations using IONM, showing a lower rate of problems as the number of procedures performed rose. They reported that the rates of problem-free use of IONM were 80 % in the first 50 operations, 92 % in the next 50, and 98 % in the last 50 (*p* < 0.05) [9]. Pragacz et al. reported an even higher rate of technical problems—24 %—in the initial period of IONM use (50 thyroid procedures); however, in the next 50 operations, the rate dropped to 8 % (*p* = 0.029) [11]. The results of the current study and those reported by other authors indicate that in order to master the basic techniques of neuromonitoring and minimize technical problems, 50 to 100 thyroid procedures with IONM have to be performed [9, 11].

Numerous publications about neuromonitoring in thyroid surgery show a high rate of RLN identification with IONM [1, 6, 13, 22–24]. At our center, effective RLN identification with IONM in the first year was quite high: 92.11 %. In the initial phase of IONM use, the main problems causing difficulty in nerve visualization were inexperience in mapping on the one hand and technical problems on the other. In the later years of the study period, along with the decrease in technical problems, there was an increase in nerve identification, by 3 % in 2013 and by another 4 % in 2014. After 3 years of working with IONM (more than 200 thyroid operations), this increase was at the level of statistical significance (*p* = 0.022). None of the patients presented postoperative palsy. Even when there was difficulty finding the nerve (R1), we always checked the vagus nerve (V2) after a lobe resection. A positive signal from the vagus nerve was particularly useful before resecting the second lobe of the thyroid in bilateral thyroid operations. Pragacz et al. noted a statistically significant increase in nerve identification (*p* = 0.006) after only 50 monitored thyroid operations and that 84 % rate increased to 99 % after the next 50 procedures [11, 19].

The question therefore arises whether we are able to significantly reduce the frequency of RLN paralysis as we learn the method of IONM. In the current study, within 3 years, there was a decline in transient paralysis from 2.63 % of the RLNs exposed to the risk of damage in 2012 to 0.83 % in 2014; this decrease, however, was not at the level of statistical significance (*p* = 0.275). Permanent paralysis occurred only in two cases (0.83 % of the RLNs exposed to the risk of damage) during the first 101 thyroid operations performed during the initial stage of IONM implementation; it did not occur at all during the remaining study period (*p* = 0.505). There was no bilateral paralysis after procedures with neuromonitoring throughout the observation period. Jonas et al. reported virtually identical results in terms of the RLN injury rate during the IONM learning period. In those authors’ observations, transient paralysis occurred in 2.3 % and permanent paralysis in 0.8 % of the RLNs at risk; as in the present study, those figures concerned only the first of 3 years working with IONM [10]. A similar proportion of transient paralysis (2.6 %) and lack of permanent injury was reported by Dionigi et al. in the first 152 thyroid surgeries using IONM [9]. On the other hand, Pragacz et al. reported a statistically significant decrease in transient RLN paralysis between the initial learning period and the team’s later operations with IONM. In those authors’ study, in the introductory period transient paralysis occurred in 6.5 % of the RLNs at risk; this was drastically reduced to 1 % in the next 50 thyroid operations [11, 19]. While it is fairly easy to learn the techniques of IONM and RLN identification, it takes a long period of observation or a very large number of thyroid procedures to observe a statistically significant drop in the RLN paralysis rate, hence the tremendous value of multicenter studies [25, 26].

A study by Dralle et al. discussing the direction of changes in thyroid surgery is worth mentioning here. The authors noted that, over the years, there have been changes in resectional strategy, with an increase in the number of total thyroidectomies and a decrease in subtotal resection, but the rates for postoperative hypoparathyroidism and vocal cord palsy have decreased [26]. Despite the relatively small number of thyroid procedures performed at our center, we also observed a statistically significant increase in the ratio of radical thyroid procedures (*p* = 0.004). There was a slight decrease in the rate of postoperative palsy, but it was not statistically significant. These results at a single small department are similar to those observed by Dralle et al. in their multicenter study. There are many different factors influencing thyroid operation strategy, but we must emphasize that at our center these results were achieved after the introduction of IONM.

Regarding the value of IONM in decreasing the number of cases of postoperative RLN paralysis, a study by Alesina et al. is worth quoting. They compared thyroidectomies performed by inexperienced surgeons under the supervision of a consultant surgeon without IONM with thyroidectomies performed without experienced oversight but with the use of IONM. The study revealed that the total number of injuries in the two groups was similar—2.6 and 2.7 %, respectively. This proved how good IONM is, offering a high degree of safety during thyroid surgery even in the hands of surgeons with little prior experience [27].

An objective assessment of the value of IONM is an analysis of its sensitivity, specificity, positive and negative predictive value, and accuracy. In the present study, the sensitivity of IONM was 71.4 % in 2012; this increased to 100 % (*p* > 0.05) after the first 101 thyroid procedures. The positive predictive value (PPV) was 62.5 % in 2012 and 75 % in the 2013–2014 period (*p* < 0.05). Despite the statistically significant reduction in the number of technical problems (*p* = 0.0185), the increases in sensitivity and PPV were not statistically significant (*p* = 0.05). These PPV values did not give us grounds to perform “stage thyroidectomies” in either period working with IONM. Pragacz et al. reported an even lower PPV in the first period of IONM use: 55.6 % after the first 50 thyroid surgeries performed [11, 19]. In the literature, positive predictive value ranges from 9.2 to 92.1 %; it was only with the standardization of neuromonitoring that we find PPV above 50 % at most centers during the introductory phase of IONM use and increasing with experience [1, 28].

In contrast, in most reports, negative predictive value (NPV) is quite high from the beginning of IONM use, ranging from 92 to 100 % [1, 8]. In the present study, NPV in the initial period was 98 %, rising to 100 % in the second stage of IONM use. However, this increase was not statistically significant (*p* = 0.05). The increase in accuracy was the only one that was statistically significant after the first 101 thyroid procedures with IONM, increasing from 97.4 % in 2012 to 99.6 % in the later phase of the study period (*p* = 0.049).

In the context of learning curves, the duration of thyroid surgery is often given, with the expectation that as the surgical team gains experience with IONM the duration of the procedures will decrease. Such a dependency was reported by Dionigi et al. and Pragacz et al. [9, 11]. The present study did not confirm a correlation between the duration of surgery and the length of time working with IONM. Rather, the operating time was different each year we worked with IONM: It was 105 min in 2012, 115 min in 2013, and 110 min in 2014 (*p* < 0.05). Every thyroid operation is different, and the procedure duration is determined by the preoperative diagnosis, the type of operation performed (primary or reoperation), and the experience and individual skills of the surgeon. Interestingly, in our analysis it emerged that the only factor that significantly influenced the time of monitored thyroid surgery was the patients’ body weight (*p* = 0.004). As the BMI increased, so did the duration of surgery, which can be explained by more difficult access to both the vagus nerve and the RLN in overweight patients.

IONM seems to be an easy technique to introduce into everyday use in clinical practice, but full exploitation of this method—among other things, the decision to undertake “stage thyroidectomy”—requires good preparation before implementation, followed by the execution of at least 50–100 thyroid procedures with IONM. Duclos et al. discussed the individuality of the learning curve, specifying how very different it was among three surgeons performing monitored thyroid surgery for the first time [20].

Finally, it must be emphasized that the surgeons’ rapid learning process with IONM would not have been possible without good cooperation with the anesthesiology team, which has also been noted by other authors [1, 8, 9].

## Conclusions

There was a sharp decrease in the number of technical problems involving equipment setup from 2012 to 2014.
